# Prevalence and clinical features of HIV and malaria co-infection in hospitalized adults in Beira, Mozambique

**DOI:** 10.1186/1475-2875-11-241

**Published:** 2012-07-26

**Authors:** Annalisa Saracino, Edy A Nacarapa, Ézio A da Costa Massinga, Domenico Martinelli, Marco Scacchetti, Carlos de Oliveira, Anita Antonich, Donata Galloni, Josefo J Ferro, César A Macome

**Affiliations:** 1 Doctors with Africa CUAMM-Mozambique, Beira, Mozambique; 2 Clinic of Infectious Diseases, University of Foggia, v.le L. Pinto 1, Foggia, 71100, Italy; 3 Catholic University of Mozambique, Beira, Mozambique; 4 Institute of Hygiene, University of Foggia-Italy, Foggia, Italy; 5 Central Hospital of Beira-Mozambique, Beira, Mozambique

**Keywords:** HIV, Malaria, Cotrimoxazole, Antiretrovirals, Mozambique

## Abstract

**Background:**

Mozambique presents a very high prevalence of both malaria and HIV infection, but the impact of co-cancel infection on morbidity in this population has been rarely investigated. The aim of this study was to describe the prevalence and clinical characteristics of malaria in hospitalized adult HIV-positive patients, treated and untreated with combination anti-retroviral therapy (ART) and cotrimoxazole (CTX)-based chemoprophylaxis, compared to HIV negatives.

**Methods:**

From November to December 2010, all adult patients consecutively admitted to the Department of Internal Medicine of Beira Central Hospital, Sofala Province, Mozambique, were submitted to HIV testing, malaria blood smear (MBS) and, in a subgroup of patients, also to the rapid malaria test (RDT). Socio-demographical and clinical data were collected for all patients. The association of both a positive MBS and/or RDT and diagnosis of clinical malaria with concomitant HIV infection (and use of CTX and/or ART) was assessed statistically. Frequency of symptoms and hematological alterations in HIV patients with clinical malaria compared to HIV negatives was also analysed. Sensitivity and specificity for RDT versus MBS were calculated for both HIV-positive and negative patients.

**Results:**

A total of 330 patients with available HIV test and MBS were included in the analysis, 220 of whom (66.7%) were HIV-positive. In 93 patients, malaria infection was documented by MBS and/or RDT. RDT sensitivity and specificity were 94% and 96%, respectively. According to laboratory results, the initial malaria suspicion was discarded in about 10% of cases, with no differences between HIV-positive and negative patients. A lower malaria risk was significantly associated with CTX prophylaxis (p=0.02), but not with ART based on non nucleoside reverse-transcriptase inhibitors (NNRTIs). Overall, severe malaria seemed to be more common in HIV-positive patients (61.7%) compared to HIV-negatives (47.2%), while a significantly lower haemoglobin level was observed in the group of HIV-positive patients (9.9±2.8mg/dl) compared to those HIV-negative (12.1±2.8mg/dl) (p=0.003).

**Conclusions:**

Malaria infection was rare in HIV-positive individuals treated with CTX for opportunistic infections, while no independent anti-malarial effect for NNRTIs was noted. When HIV and malaria co-infection occurred, a high risk of complications, particularly anaemia, should be expected.

## Background

Since 2009, the CDC has included malaria in the list of AIDS-related opportunistic infections [1]; even though malaria is not among the leading causes of death in HIV-infected patients [2], it has been identified as the third most important source of HIV-related morbidity in Africa [3]. In fact, HIV infection is expected to increase the morbidity and mortality attributed to malaria, since immuno suppression impairs the immune response to Plasmodium, determining more frequent occurrences of clinically severe malaria [4]. However, the use of cotrimoxazole (CTX) prophylaxis and antiretroviral therapy (ART) in HIV-infected patients seems to provide a protective effect from malaria [4-6].

In Mozambique, all regions show a very high prevalence of both malaria and HIV infections, particularly Beira (Sofala province), which is a historically important harbour on the Indian ocean, situated at the beginning of the commercial route to Zimbabwe and central Africa. In fact, HIV prevalence is approximately estimated at 30% in the urban area of Beira [7,8], while malaria transmission is stable throughout the country with peaks from December to April. *Plasmodium falciparum* accounts for >95% of cases at a rate of about 200 per 1,000 inhabitants (all ages) in 2009. While coverage with an artemisin-based combination therapy (artemether-lumefantrine), adopted in 2004, is adequate, the distribution of insecticide-treated nets and indoor residual spraying have remained low (43% and 37%, respectively, in 2009) [9]. On the other hand, the introduction of ART in Mozambique initiated in 2004, has reached a coverage of around 40% for patients with a CD4 level<200 cells/mmc in 2008 according to UNAIDS estimations [8]. Therefore, the real impact of therapy implementation, along with the use of CTX-based prophylaxis on mortality and morbidity due to AIDS-related opportunistic infections, including malaria, still needs to be fully evaluated.

Moreover, the frequent overlap between clinical signs and symptoms of HIV and malaria, especially regarding fever and anaemia, can determine many difficulties for diagnosis. A previous study in Mozambique [10] demonstrated a statistically significant association between HIV status and risk of receiving an incorrect malaria diagnosis. In fact, according to recent WHO guidelines, the confirmation of diagnosis by microscopy (malaria blood smear, MBS) or rapid diagnostic tests (RDTs) is recommended for all patients with suspected malaria before treatment is initiated, but presumptive treatment is still a common practice in malaria-endemic resource-limited settings. According to the last WHO report (2010) regarding malaria in Mozambique, only 13% of cases were confirmed by microscopy and/or RDT [9].

The aim of this study was to describe the prevalence and clinical characteristics of malaria infection in hospitalized adult HIV-positive patients treated and untreated with ART and CTX, compared to HIV-negatives.

## Methods

From November to December 2010, all adult patients (> 15 years, according to the hospital policy) consecutively admitted to the Department of Internal Medicine of the Beira Central Hospital, Sofala, Mozambique were enrolled in the study.

The main objective of the study was to verify the association of a malaria infection with the contemporary presence of HIV infection, treated or untreated by CTX and ART. For this purpose, all patients with a positive malaria blood slide (MBS) and/or rapid diagnostic test (RDT) were considered as infected with malaria.

The secondary objectives were:

to evaluate the accuracy of RDT in both HIV-negative and positive patients

to compare the frequency and severity of clinical symptoms and the hematological alterations in patients with concurrent clinical malaria and HIV with respect to HIV seronegatives. Patients for whom, despite a positive malaria test, a diagnosis of clinical malaria was considered not plausible by clinicians based on clinical history (absence of fever at admission or in the previous 24 hours and/or evidence of symptoms and signs, e.g. neck stiffness, suggestive of other diagnoses and confirmed by microbiological and/or histological data) were excluded from this sub-analysis.

According to the hospital policy, voluntary counseling and testing for HIV was offered to all admitted patients by a trained nurse, and performed in all cases of acceptance, with the exception of those who were already ascertained HIV-positive. HIV tests were performed with both the Determine (Abbot Japan co, Ltd) and Unigold tests (Trinity Biotech plc, Bray, Ireland).

During the study period for purposes of analysis, a malaria blood slide (MBS) was obtained from all patients consecutively hospitalized in Internal medicine, even in the absence of highly suspicious symptoms such as fever. malaria slides were prepared and evaluated as part of the hospital routine. Parasitaemia was assessed using a semi-quantitative method providing a score as follows: 1 (+=1–10 parasites per 100 fields); 2 ( ++=11–100 parasites per 100 fields); 3 (+++=1–10 parasites per 1 field ); 4 (++++=more than 10 parasites per field). A RDT (Malaria P.F. HRP II Ag rapid test, Orchid Biomedical Systems,goa, India) was also performed in a subgroup of 160 cases, consecutively enrolled during the first month of the study period.

Socio-demographical and clinical data were collected by two fifth-year medical students from the Catholic University of Mozambique, Beira, under the supervision of the researchers and attending clinicians. Information included the diagnosis at admission formulated by the first visiting medical physician, the main symptom/signs referred at time of admission based on data obtained by questions to patients and on direct clinical examination. The available blood parameters included white blood cell count (number/mmc), platelet count (number x 100 000/mmc) and haemoglobin (mg/dl). Blood glucose level (mmol/L) was available only in a minority of cases. The results of HIV and malaria testing were recorded for all patients. For the HIV-positive patients, the date of the first positive anti-HIV test, the use of CTX as chemoprophylaxis for opportunistic infections, and the use and date of ART initiation were also reported. The CD4 cell count was available only in a minority of patients and was not considered in the analysis. HIV-RNA measurement was not available in the hospital at time of study.

Based on available laboratory parameters, clinical malaria was classified as complicated or severe according to the presence of neurological symptoms (impaired consciousness or multiple convulsions), severe dyspnea or radiological signs of pulmonary edema, hypotension (systolic pressure<70mmhg), severe anaemia (<5mg/dl), severe jaundice, spontaneous bleeding, haemoglobinuria, oliguria and, when available, hypoglycaemia (<2.5mmol/L). Parasitaemia was not included among the parameters as only a semi quantitative method was furnished by the laboratory.

The study was approved by the Ethical Committee of the Beira Central Hospital. Data were obtained in the context of normal clinical routines. However, all patients provided oral informed consent for the use of their data for research purposes. For unconscious patients, the family members were asked to give consent.

### Statistical analysis

Descriptive statistics were produced for the demographic and clinical characteristics of all cases. The mean and SD are presented for normally distributed variables. The Student t test (Kruskal-Wallis rank sum test or mann–Whitney median test for skewed distributions) was used to compare quantitative variables, and the Pearson chi square test (Fisher exact test where appropriate) was used for categorical variables. Univariate and multivariate analysis by means of logistic regression were carried out to investigate variables associated with a positive malaria test. A p value<0.05 was considered statistically significant. All analyses were performed with EpiInfo software, version 3.5.3 (CDC, Atlanta).

## Results

A total of 342 adult patients admitted to the Department of Internal medicine of the Beira Central Hospital were enrolled in the study, 330 of whom (mean age of 38±15years, 50.9% females) had an available HIV test and malaria blood slide and were included in the analysis. Twelve patients refused to be submitted to HIV testing and were excluded from the study; the demographic and clinical characteristics of these patients did not significantly differ from those of the included patients (data not shown). The characteristics of patients enrolled in the study according to the results of HIV and malaria testing are summarized in Table 1.

**Table 1:** Patient characteristics

		**HIV**			**Malaria**		
	**Total**	**Positive**	**Negative**	***p value***	**Positive**	**Negative**	***p value***
**No. patients** (%)	330 (100%)	220 (66.7%)	110 (33.3%)		93 (28.2%)	237 (71.8%)	
**Gender Female**	168 (50.9%)	111 (50,5%)	57 (51.8%)	***0.4***	68 (73.1%)	100 (42.2%)	***<0.001***
**Age** (years,mean±SD)	38±15	35±11	43±19	***0.002***	34±14	40±15	***0.001***
**HIV known**		104 (47.3%)	-				
**CTX prophylaxis**		78 (35.4%)	-				
**ART**		69 (31.4)	-				

A total of 220/330 (66.7%) enrolled patients resulted HIV-positive, but only 104/220 (47.3%) were already aware of their HIV-seropositivity before admission; in the remaining 116 cases, diagnosis of HIV infection was performed for the first time during hospitalization. HIV-positive patients were equally distributed for gender (50.4% females), while the mean age was significantly lower among HIV-infected patients compared to HIV-negatives (35.5±11.9 versus 43.4±19.5, respectively; p=0.002). A WHO stage III-IV was presented by 77% (140/182 available) of patients.

Daily CTX chemoprophylaxis was being administered to 78/220 (35.4%) HIV-positive patients at time of admission, while only 69 patients (31.4%) were assuming antiretroviral therapy, 55 of whom were concurrently receiving CTX. All patients, except two, were treated by an ART based on NNRTIs (nevirapine in 63 cases and efavirenz in 4 cases); the NRTI backbone was composed of a stavudine and lamivudine combination in 42 patients and by zidovudine and lamivudine in the remaining 12. Only two patients were assuming a second-line regimen represented by two previously unused NRTIs and lopinavir/ritonavir. The median duration of ART was four months (range 1–48).

Among the 330 patients consecutively subjected to a malaria blood slide, 90 (27.3%) resulted positive. In a subgroup of 160 patients, consecutively admitted, a rapid diagnostic test for malaria was also performed resulting in 50 positives; discordant results were obtained in only seven patients, three of whom presented a positive RDT despite a negative blood slide, while RDT yielded negative results despite a positive MBS in four patients. Therefore, when considering the blood slide as a gold standard, the RDT sensitivity was 94% with a specificity of 96%, with no remarkable differences between the two groups of HIV-positive and negative patients. The group of patients with a malaria infection included a higher number of females (68/93, 73.1%, p<0.001) and younger patients (mean age 34.3±14.2 versus 39.6±15.5 in malaria-negatives, p=0.001). For purposes of analysis, all patients (n=93) presenting a positive MBS and/or RDT were considered as infected with malaria parasites.

Clinicians had previously indicated malaria as the main reason for hospital admission in a total of 86/330 cases (26.1%). When data were retrospectively analysed according to results of HIV testing, it was observed that malaria appeared to be the most frequently presumed reason (33.6%) for admission in the group of HIV-negative patients, while clinical suspicion of tuberculosis was the main reason for admission in the group of HIV-positive patients (32.3%), followed by malaria in 22.6% of cases. The hypothesis of malaria was rejected, based on laboratory results, in 9/86 (10.5%) patients for whom a clinical suspicion had been raised, 6/49 (12%) of whom resulted HIV-positive and 3/37 (8.1%) HIV-negative, without any difference between the two groups (p=0.7).

Overall, the frequency of a positive malaria test was slightly lower among HIV-positives compared to HIV-negative patients (25.9% versus 32.7%, respectively), without any statistically significant difference. The diagnosis of malaria was significantly less likely in the group of patients assuming a daily cotrimoxazole chemoprophylactic regimen compared to those without prophylaxis (12.8% versus 32.9%, respectively, p<0.001) and in patients assuming ART compared to untreated patients (17.4% versus 29.8%, respectively, p=0.03). At multivariate analysis, however, only CTX prophylaxis (p=0.02), together with age ≥35years (p=0.004) and male gender (p<0.001), were independently associated with a lower risk of malaria infection (Table 2).

**Table 2:** Univariate and multivariate analysis of factors influencing probability of positive malaria test (malaria blood slide and/or rapid diagnostic test, n=93)

	**OR (95% I.C.)**	***p value***
**Univariate analysis**		
Gender (M vs F)	0.27 (0.16-0.45)	*<0.001*
Age (≥35 vs<35)	0.44 (0.27-0.72)	*<0.001*
HIV infection (yes vs no)	0.71 (0.43-1.18)	*0.100*
Cotrimoxazole prophylaxis (yes vs no)	0.20 (0.14-0.61)	*<0.001*
Antiretroviral treatment (yes vs no)	0.49 (0.24-1.01)	*0.030*
**Multivariate analysis**		
Gender (M vs F)	0.29 (0.17-0.51)	*<0.001*
Age (≥35vs<35)	0.46 (0.27-0.77)	*0.004*
HIV infection (yes vs no)	0.84 (0.47-1.5)	*0.568*
Cotrimoxazole prophylaxis (yes vs no)	0.31 (0.11-0.84)	*0.022*
Antiretroviral treatment (yes vs no)	1.18 (0.44-3.15)	*0.735*

The level of parasitaemia was slightly lower in HIV-positive versus HIV-negative patients (mean 1.8±1.1 versus 2.3±1.2, respectively, p=0.08), but when patients on CTX and/or ART were excluded, no significant differences were observed between the two groups (mean 2.3±1.2 versus 2.0±1.2, respectively, p=0.2).

Based on patient history and clinical examination, clinical malaria was excluded for 10/93 patients with a positive malaria test, six of whom because of complete absence of fever and four because of a diagnosis of bacterial meningitis, suspected on the basis of neck stiffness and ascertained by liquor examination after rachicentesis. These excluded ten patients presented a positive mBS with low grade parasitemia (only one cross positivity in four cases and two crosses in the additional four patients), which was assumed to be premunity, without relevance for the patient’s clinical condition at time of admission; another two patients were RDT positive/slide negative. Regarding distribution of clinical malaria, a higher prevalence was observed in the group of HIV-negative patients (36/330, 32.7%) compared to HIV-positives (47/330, 21,4%, p=0.01). However, when patients on CTX chemoprophylaxis and/or ART were excluded, the proportion of HIV-infected patients admitted for clinical malaria was similar to that of HIV-negative subjects (42/238, 32.8%, p=0.54).

Clinical and laboratory characteristics of the 83 patients with clinical malaria were then compared in the HIV-positive and HIV-negative groups (Table 3). Overall, severe malaria seemed to be more common in the group of HIV-positive patients (61.7%) compared to HIV-negatives (47.2%) but this difference was not statistically significant (p=0.13). Among the reported symptoms and signs, a higher frequency of dyspnea (p=0.09) and diarrhea (p=0.03) was observed among HIV-positive patients, while headaches (p=0.03) and hypotension (p=0.05) were more frequently found in HIV-negative patients. No differences between the two groups were reported regarding nausea, vomiting, jaundice, cough, weight loss and neurological symptoms, including convulsions and coma. Fever was not included in this list because it was considered a criteria for defining clinical malaria in both groups. Furthermore, no differences were observed between the two groups regarding percentage of patients for whom a hepato-splenomegaly was reported.

**Table 3:** Clinical characteristics and blood parameters in 83 patients diagnosed with clinical malaria according to HIV serology

	**HIV positive**	**HIV negative**	***p value***
	**No. (%)**	**No. (%)**	
**Patients with severe malaria**	29 (61.7%)	17 (47.2%)	*0.13*
**Clinical symptoms and signs**			
Headache	35 (74.5%)	33 (91-7%)	*0.03*
Jaundice	10 (21.3%)	5 (13.9%)	*0.28*
Cough	17 (36.2%)	10 (27.8%)	*0.28*
Dyspnea	15 (31.9%)	6 (16.7%)	*0.09*
Nausea	33 (70.2%)	26 (72.2%)	*0.51*
Vomiting	33 (70.2%)	26 (72.2%)	*0.40*
Diarrhoea	19 (40.4%)	7 (19.4%)	*0.03*
Hypotension	0	6 (16.7%)	*0.05*
Convulsions and/or coma	7 (14.9)	6 (16.7)	*0.52*
Weight loss	20 (42.6%)	11 (30.6%)	*0.18*
Hepatomegaly	22 (46.8%)	14 (38.8%)	*0.30*
Splenomegaly	22 (46.8%)	11 (30.6%)	*0.10*
**Blood parameters**	mean±SD	mean±SD	
**WBC**/mmc	7.7±2.5	7.7±3.7	*0.25*
**Hb**mg/dl	9.9 ±2.8	12.1±2.8	*0.003*
**PLT** (x100.000/mmc)	188.4±120.9	218.8±126.1	*0.07*
**Glycaemia** (mmol/L)*	4.8±1.5	7.4±1.6	*0.02*

*data available only for 17 patients.

The following laboratory parameters were available for comparison: white blood cells (WBC), haemoglobin (Hb), platelets (PLT) and glycaemia. The total number of WBC was comparable between HIV-positive and negative patients with malaria (mean 7.7±2.5 versus 7.7±3.7 cells/mmc, respectively, p=0.2). On the contrary, a lower Hb level was observed in the group of HIV-positives (9.9±2.8mg/dl) compared to HIV-negative subjects (12.1±2.8mg/dl) (p=0.003) along with an inferior platelet count (188.4±120.9 cells/mmc versus 218.8±126.1 cells/mmc, respectively, p=0.07). A lower level of Hb (8.4±3.7 vs 9.6±2.8g/dl, p=0.006) was observed in the group of HIV-positive patients treated with ART, along with an higher prevalence of both severe (Hb<7g/dl) and moderate (Hb<10g/dl) anemia. A total of 6/12 patients treated by AZT presented moderate anemia.

Glycaemia was measured only in a small percentage of malaria patients (17/83); a lower level of glucose was recorded among HIV patients (4.8±1.5mmol/L versus 7.4±1.6, p=0.01).

## Discussion

Mozambique is a country with stable *P. falciparum* transmission, where malaria-related morbidity and mortality is still quite frequent, with more than four million cases and 4,000 attributed deaths reported each year [9]. The study was performed during two high-risk months for malaria; in fact, transmission is seasonal, mainly between November and July with peaks from December to April in the Central region of the country.

At the same time, the proportion of individuals coexisting with HIV in the whole population is about 16% according to the last epidemiological survey in 2007 [7], with the Northern and Central regions presenting a higher prevalence compared to the Southern part of the country. In particular, the Sofala province shows a prevalence rate between 17% and 33%, with the highest peaks involving urban areas. Among these, Beira, which is the 2nd largest city in Mozambique (population of more than 400,000 inhabitants), has one of the highest adult HIV seroprevalence rates in the country. The national survey based on sentinel posts throughout the country shows that the rate of HIV prevalence measured in pregnant women reached a peak of 36% in Beira in 2002, stabilizing around 29% in the last report in 2007 [7] while the 2009 WHO report gives a range between 25% and 39.9% for Beira [8]. This rate is extra ordinarly high and is probably due to geographical and historical reasons linked to its role as a key commercial harbor on the Indian Ocean, thus probably favouring a high sexual promiscuity. This is reflected in the impressive HIV prevalence (66.7%) in patients hospitalized in the Internal medicine Clinic of the Central Hospital of Beira serving a wide area of Central Mozambique.

Only a few studies have evaluated the epidemiological and clinical aspects concerning concurrent HIV and malaria in this country [10] and, none in the above-mentioned area. In particular, data are lacking on the reciprocal influence of the two infections on the morbidity of the Mozambican population following the contemporary introduction in 2004 of both ART for HIV infection and a new artemisinin-based treatment for malaria.

Malaria, according to our analysis, is still the most frequent cause of adult admissions to Internal Medicine wards in Mozambique (>25% of all motives for hospitalization). However, when evaluating patients based on their HIV serological status, results show that, although HIV-infected patients are considered a high risk group for malaria, malaria is not the most frequent diagnosis in this patient group, and the prevalence of both malaria infection and clinical malaria is lower among HIV-infected patients (26% and 21%, respectively) than among HIV-negatives (33% and 32%, respectively). This might imply at least two different explanations: The first is that, naturally, the prevalence of malaria infection in hospitalized patients does not directly reflect the disease distribution throughout the entire population, due to the greater number of pathologies, especially AIDS-related opportunistic infections, which could require hospitalization in HIV-infected individuals compared to HIV-negatives. In fact, a previous study performed in Maputo, Mozambique, has already evidenced that the fraction of febrile illness attributable to malaria is lower in HIV-positive adults than in HIV-negatives [10]. However, a second explanation is possible as a statistically significant and independent association between CTX use and decreased risk of malaria infection was observed, while, on the contrary, the malaria distribution did not differ between untreated-HIV-positive and HIV-negative patients. These results are not surprising as the protective effect of cotrimoxazole on parasite infection has been well-documented in clinical trials in children [11], pregnant women [12] and adults [6,13]. The present study confirms this protective effect also in the “real word” scenario of an African urban hospital, outside the selected conditions of a randomized clinical trial.

The effect of anti-virals on malaria parasitaemia is still questionable [4,5], and only protease inhibitors are currently considered to provide a proven anti-malarial efficacy [14-19], even if in a recent study, a low anti genaemia was demonstrated for suppressed HIV-infected patients treated with ART and CTX for long periods [20]. In this study it was not possible to individuate aviremic patients, because neither viral load nor CD4 count were available; however, the median duration of ART was only four months and 77% of patients were classified as WHO stage III-IV. The majority (67/69) of treated patients were on NNRTIs; an association between the use of antiretrovirals and lower risk of malaria was observed at univariate analysis but it most likely depended on the protective effect of CTX rather than on the direct impact of ART, as statistical significance was not confirmed with multivariate analysis. Hence, further studies directly assessing this topic are required to validate this association.

A wider implementation of prophylaxis for HIV-infected subjects in sub-Saharan Africa in future years might, therefore, substantially reduce the burden of malaria infection and consequently its morbidity in these patients. Moreover, it also appears that the problem of a greater access to HIV testing is the fundamental issue. In fact, the extensive use of HIV testing in the hospital demonstrates that more than one-half of HIV-positive subjects were unaware of their seropositivity before hospitalization. Thus, among HIV-infected patients, only 31% and 35% were already receiving antiretroviral therapy and/or chemoprophylaxis for opportunistic infections, respectively. Moreover, low rates of regimen switches were described (only two patients were on a second line regimen): this is not surprising as the average ART-treatment time in the present study was only four months; moreover the lack of testing for viral load renders it difficult to early individuate therapy failure. These data are consistent with the study of Auld et al. [21] on ART implementation in Mozambique, even if in disagreement regarding the rate of prescription of CTX, which was lower in their survey compared to ours.

Despite the low frequency of infection and clinical malaria diagnosed in HIV-positive patients, no statically significant differences were noted either in parasitaemia or in occurrence of severe malaria, but this study presents two limitations: the use of a semi quantitative method for measuring parasitaemia whose low accuracy does not permit us to deduct any definite conclusions, and the lack of many laboratory parameters which render incomplete our criteria for classification of complicated malaria. However, a trend toward a more severe presentation characterized the HIV-malaria co-infection, with more frequent symptoms, such as dyspnea and diarrhea, occurring in the HIV population. Remarkably, anaemia was significantly associated with HIV-infection in malaria patients, and a trend towards a lower platelet number was also observed in co-infected individuals. Cytopenia of all major blood cell lines have been recognized in patients with chronic HIV since the beginning of the epidemic [22] and its incidence directly correlates with the degree of immuno suppression, although isolated abnormalities, particularly thrombocytopenia, may be encountered also as the initial presentation of HIV infection. In this analysis, anaemia was also associated with use of ART. ART has been demonstrated to improve preexisting anemia after use for at least 6 months [23], but the median length of therapy in our series was only four months. Therefore, the association between anaemia and ART probably only indicates a more advanced stage of HIV infection in treated patients. These results confirm and extend previous observations [24] emphasizing the need to avoid the dual infection, thus limiting hematological complications in these patients.

Lastly, the study was carried out in a 3^rd^ level hospital where blood slides for confirming malaria have been largely used for some years; for the purposes of the analysis, MBSs were utilized for all hospitalized patients in Internal medicine, thus permitting us to reject about 10% of the presumptive malaria diagnoses; contrary to previous observations [10], an overestimation of malaria in HIV-positives when compared to HIV-negatives was not noted. The results of RDT were comparable to those of MBS in both HIV-infected and non-infected patients, independently of simultaneous treatment with ART; RDT use did not seem to provide an added value for correct diagnosis in patients already submitted to MBS, but the high sensitivity (94%) and specificity (96%) obtained in our survey confirmed that they are suitable in all fields in which MBS is not feasible and accuracy cannot be assured due to lack of expertise.

## Conclusions

In conclusion, malaria infection appears to be rare in HIV-infected individuals when treated with a cotrimoxazole-based chemoprophylaxis for opportunistic infections, even in an area with a high co-infection rate such as Mozambique; therefore, a more extensive use of CTX for opportunistic infections in malaria endemic countries is warranted. No independent effect of NNRTIs on malaria infection was evidenced in our study. When HIV and malaria co-infection occurs, a high complication risk, particularly anaemia, should be considered.

## Abbreviations

ART, Antiretroviral Therapy; CTX, Cotrimoxazole; MBS, Malaria Blood Slide; RDT, Rapid Diagnostic Test; NRTIs, Nucleoside/Nucleotide Reverse-Transcriptase Inhibitors; NNRTIs, Non nucleoside Reverse-Transcriptase Inhibitors; PIs, Protease Inhibitors; WHO, World Health Organization.

## Competing interests

The authors declare that they have no competing interests.

## Authors' contributions

AS and MS conceived of the study and participated in its design and coordination; AS drafted the manuscript. EAN, EACM, CdO and CM were responsible for data collection and participated to the study design. DM performed the statistical analysis. AA, DG, JF participated in the study coordination, interpretation of data and revised the paper critically. All authors read and approved the final manuscript.
